# Characterization of a Novel Esterase Est33 From an Antarctic Bacterium: A Representative of a New Esterase Family

**DOI:** 10.3389/fmicb.2022.855658

**Published:** 2022-05-17

**Authors:** Xiaoyu Liu, Mingyang Zhou, Rui Sun, Shu Xing, Tao Wu, Hailun He, Jianbin Chen, John Kevin Bielicki

**Affiliations:** ^1^School of Chemistry and Chemical Engineering, Qilu University of Technology (Shandong Academy of Sciences), Jinan, China; ^2^State Key Laboratory of Medical Genetics, School of Life Sciences, Central South University, Changsha, China; ^3^Shandong Provincial Key Laboratory of Molecular Engineering, Qilu University of Technology (Shandong Academy of Sciences), Jinan, China; ^4^Lawrence Berkeley National Laboratory, University of California at Berkeley, Berkeley, CA, United States

**Keywords:** esterase, Antarctic, bacterium, soil, new esterase family XXI

## Abstract

Studies of microorganisms from extreme environments can sometimes reveal novel proteins with unique properties. Here, we identified a novel esterase gene (*Est33*) from an Antarctic bacterium. The protein was expressed and purified for biochemical characterizations. Site-mutation variants including S94A, D205A, and H233A were constructed to explore the structure–function relationship of the catalytic triad of Est33, and we found mutating Ser^94^, Asp^205^, and His^233^ residues lead to a complete loss of enzyme activity. In addition, the catalytic Ser^94^ located in a conserved pentapeptide motif GVSWG. Phylogenetic analysis showed that Est33 and its closely related homologs belonged to an independent group apart from other known family members, indicating that Est33 represented a new family of esterase. The Est33 enzyme was found to be a cold-active esterase retaining 25%–100% activity from 10°C to 30°C and to have optimal catalytic activity toward *p*-nitrophenol acetate (30°C and pH7.5). The serine modifying reagent phenylmethylsulfonyl fluoride inhibited the activity of Est33 by 77.34%, while thiol reagents such as dithiol threitol (DTT) activated the enzyme by 3-fold. Metal chelating reagents EDTA had no effects, indicating that Est33 is not a metalloenzyme. Collectively, these results indicate that Est33 constitutes the first member of a novel esterase family XXI that has been identified.

## Introduction

Lipolytic enzymes are a class of biocatalysts that generally have broad substrate specificity, chiral selectivity, and tolerance to organic reagents. Consequently, these enzymes play an important role in many biotechnological applications (Rong et al., 2018). Esterase (EC3.1.1.1) and lipase (EC3.1.1.3) are both lipolytic enzymes, which can catalyze the cleavage and formation of ester bonds. Most of enzymes belong to the α/β hydrolase superfamily and share a canonical GXSXG-pentapeptide sequence around the catalytic serine (Jochens et al., 2011; Kovacic et al., 2018). In general, esterases catalyze the hydrolysis of short-chain ester substrates and follow classical Michaelis–Menten kinetics. These features distinguish esterases from lipases, which typically hydrolyze water-insoluble long-chain acyl substrates and exhibit interfacial activation (Bornscheuer, 2001). However, interfacial activation was not unsuitable for the classification of lipase and esterase, because some lipases did not exhibit such phenomenon, such as Lip4 from *Candida rugosa* (Tang et al., 2001) and lipase-B from *Candida antarctica* (Uppenberg et al., 1994).

Many lipolytic enzymes from bacteria have been reported in recent years. Based on the amino acid sequence and biological characteristics, Arpigny and Jaeger first classified lipolytic enzymes into eight (I–VIII) families (Arpigny and Jaeger, 1999). Subsequently, Handrick et al. (Handrick et al., 2001) discovered the lipase-like poly [(R)-3-hydroxybutyrate] depolymerase PhaZ7 and classified it into a new family IX. The lipolytic family of enzymes has since been expanded with the discovery of new members such as EstD, LipEH166, EstA3, EstDZ2, estUT1, and Est903 (Levisson et al., 2007; Kim et al., 2009; Rao et al., 2011; Zarafeta et al., 2016; Samoylova et al., 2018; Jia et al., 2019). Recent classifications by Kovacic et al. (Kovacic et al., 2018) and Wang et al. (Wang et al., 2020) resulted in the 19th and 20th families, respectively.

Lipolytic enzymes can also be found in organisms living in extreme environments, such as high- (Chow et al., 2012) and low- temperatures (Tchigvintsev et al., 2015) and elevated salt concentrations (Sood et al., 2018). Ester hydrolases isolated from these organisms are evolutionarily adapted to different types of environmental conditions (De Luca and Mandrich, 2020). For example, esterase Est1 isolated from bacteria of soil from hot springs is thermostable at temperatures of approximately 70°C (Tirawongsaroj et al., 2008) while, in contrast, esterase Lip3 isolated from Arctic sediments exhibits cold adaptability and salt tolerance (De Santi et al., 2016). In addition, esterase estOKK isolated from deep-sea hydrothermal vents is alkalistable (Yang et al., 2018). These unique properties enable a wide-range of industrial- and biotechnology-applications for the lipases and esterases described.

Here, we identified a novel esterase Est33 from *Pseudomonas* sp. E5-12, which was isolated from Antarctic soil (Liu et al., 2021a). The *est33* gene was cloned and overexpressed in *E. coli*. The biochemical characteristics of this new enzyme are described. Est33 was a cold-active esterase and had optimal catalytic activity toward *p*-nitrophenol acetate at 30°C and pH7.5. Sequence and phylogenetic analysis suggested that Est33 represents a new esterase family, designated as family XXI.

## Materials and Methods

### Bioinformatic Analysis

The presence of a putative signal sequence was predicted using SignalP server 5.0 (Almagro Armenteros et al., 2019). SWISS-MODEL server^1^ was used to predict the structure of Est33 and its homologs (Waterhouse et al., 2018). ClustalX (Jeanmougin et al., 1998) and ESPript 3.0 were used to align sequences (Robert and Gouet, 2014). Comparing to reference amino acid sequences that retrieved from GenBank and the Uniprot database, the Maximum Likelihood phylogenetic tree was constructed using the IQ-TREE webserver with the auto substitution model (Trifinopoulos et al., 2016) and then modified by iTOL v6 (Letunic and Bork, 2021). The GenBank accession number of the *est33* gene was MZ717198.

### Purification of Est33 and Its Variants

The primer synthesis and DNA sequencing were performed commercially at BioSune Company (Shanghai, China). The *est33* gene was amplified from the genome of *P*. sp. E5-12 with primers est33-F and est33-R listed in Supplementary Table S1. Variants containing S94A, D205A, and H233A point substitutions were constructed by site-directed mutagenesis. The purified PCR product was ligated into the pET-22b vector (Novagen, United States) with *Hind*III and *Nde*I using NovoRec® plus One-step PCR Cloning Kit (Novoprotein, China). The *E. coli* BL21 (DE3) cells were transformed with constructed pET22b-*est33*, pET22b-*est33*^S94A^, pET22b-*est33*^D205A^, or pET22b-*est33*^H233A^ vectors and further verified by DNA sequencing.

For protein expression, *E. coli* BL21 (DE3) containing recombinant plasmid was cultured in LB medium supplemented with 100 ug/ml ampicillin at 37°C. When the OD_600_ reached the range of 0.6–0.8, isopropyl-β-D-thiogalactopyranoside (IPTG) was added at a final concentration of 0.5 mM to induce the expression of the target protein, and then, the cells were further cultivated at 17°C, 120 rpm for 48 h. The induced cells were collected by centrifugation at 10,000 × *g* for 10 min (4°C) and washed three times using Tris-buffered saline (50 mM Tris–HCl and 100 mM NaCl, pH 8.0). After disrupting cells by sonication and centrifugation at 11,000 × *g* for 30 min at 4°C, a crude enzyme solution (i.e., supernatant) was obtained. It was then passed over a Ni-Sepharose Fast Flow resin (GE Healthcare, United States) against the C-terminal 6*His tag to purify the esterase protein. The protein was recovered with an elution buffer (50 mM Tris–HCl, 100 mM NaCl, and 50 mM or 250 mM imidazole, pH 8.0), and then was dialyzed using 50 mM Tris–HCl (pH 8.0) to remove NaCl and imidazole. Purity of the resulting protein was estimated by sodium dodecyl sulfate-polyacrylamide gel electrophoresis (SDS-PAGE). N-termini protein sequencing was performed by a commercial company (Biotech-Pack, China).

### Esterase Activity Assay

Protein concentration of enzyme samples was determined by the Bradford method, with bovine serum albumin (BSA) as a standard (Kielkopf et al., 2020). Activity of the purified esterase was determined as previously described (Liu et al., 2021b). Stocking solutions of various *p*-nitrophenyl ester substrates (*p*NPs), including acetate (C2), butyrate (C4), caproate (C6), caprylate (C8), caprate (C10), laurate (C12), and palmitate (C16), were prepared in 2-propanol to a concentration of 10 mM. The standard reaction system contained 0.02 ml of 10 mM *p*NP, 0.96 ml of 50 mM Tris–HCl buffer (pH 7.5), and 0.02 ml of enzyme. After incubation at 30°C for 10 min, 0.1 ml of 20% SDS (w/v) was added to stop the reaction. The esterase activity was calculated from the release of *p*-nitrophenol measured by the OD_405_. One unit of enzymatic activity was defined as 1 μmol of *p*-nitrophenol released per minute. The standard reaction system with 0.02 ml of 50 mM Tris–HCl buffer (pH 7.5) instead of enzyme was used as the blank control.

### Enzyme Characterization and Kinetic Parameters of Est33

The substrate specificity of Est33 was determined with *p*NP esters. To evaluated the optimum temperature and pH, *p*NPC2 was as the substrate, with a temperature ranging from 0°C to 70°C (10°C or 5°C interval) and a pH ranging from 4 to 10 using Britton–Robinson buffer at 30°C. To explore the enzyme thermal stability, Est33 esterase was incubated at 30°C, 40°C, 60°C, and 90°C for 120 min, and the residual activity was determined every 15 min.

The kinetic assays were performed using *p*NPC2 as the substrate at a final concentration of 0.01–4.0 mM in 50 mM Tris–HCl buffer (pH 7.5) at 30°C. The kinetic parameters including *K*m, *V*max, and *k*cat were calculated by curve fitting of the Michaelis–Menten equation, using GraphPad Prism (version 8.0.2, La Jolla, CA). The catalytic efficiency of Est33 for *p*NPC2 was calculated as *K*m/*k*cat.

### The Effect of Additives on Est33 Activity

The effects of potential inhibitors, organic solvents, and metal ions on Est33 activity were evaluated using reaction mixtures described above (section Esterase Activity Assay) with the *p*NPC2 substrate. The potential inhibitors PMSF, EDTA, and DTT were tested at 1 mM and 10 mM final concentrations, while SDS and Tween-80 were tested at 1 and 10% (w/v), respectively. Various metal ions including Li^+^, K^+^, Mg^2+^, Ca^2+^, Mn^2+^, Fe^2+^, Co^2+^, Ni^2+^, Cu^2+^, and Zn^2+^ were tested at concentrations of 1 and 10 mM. The effects of different organic solvents were evaluated by addition of methanol, formaldehyde solution, ethanol, acetonitrile, acetone, isopropyl alcohol, and dimethyl sulfoxide to final concentrations of 20% and 40%. Enzyme activity without any additives was set at 100%. The experiment was performed at 30°C for 10 min.

## Results

### Sequence Analysis

**Figure 1:**
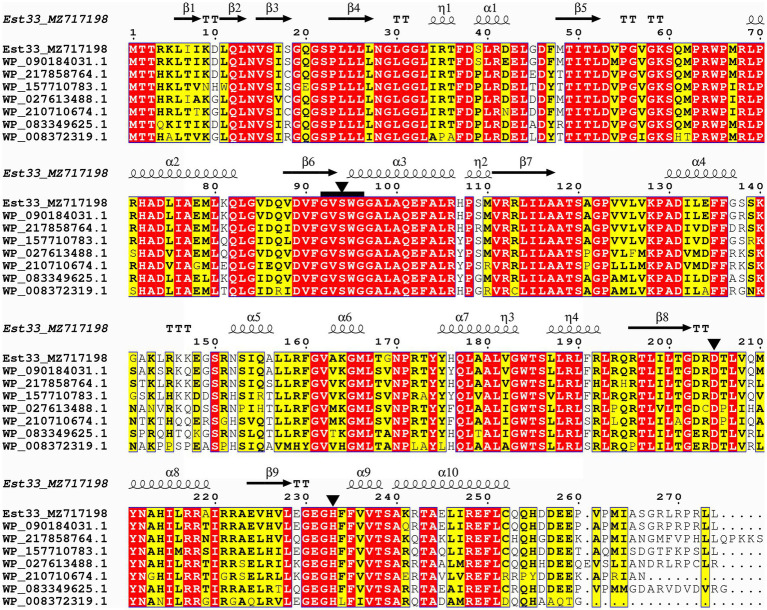
Sequence alignment of Est33 and its homologs. White letters on a red background indicate identical residues, while black bold characters and yellow box indicate similar residues. Secondary structures of Est33 are shown above the alignment. Helices, strands, turns, and 3_10_ helices are indicated by springs, arrows, TT letters, and η letters, respectively. Residues forming the catalytic triad Ser, Asp., and His black are marked by triangle. The conserved pentapeptides GXSXG are marked by black squares. The amino acid sequences used were α/β hydrolases annotated from *P. arsenicoxydans*, *P*. sp. SWRI79, *P*. sp. PB120, *P. umsongensis*, *P*. sp. URIL14HWK12:I6, *P. koreensis*, and *P*. sp. M47T1, and the identity with Est33 is 94.5%, 86.5%, 81.4%, 77.7%, 74.8%, 82.9%, and 72.9%, respectively.

We found that the *est33* gene contains an open reading frame of 825 bp, which encodes a protein of 274 amino acids. The theoretical molecular weight of the protein was 30.6 kDa. No signal sequence was detected in Est33. Protein BLAST analysis at NCBI revealed that the first 100 hits with highest sequence identity were all uncharacterized α/β hydrolases annotated from the bacterial genome. Among them, seven sequences with different identities (72.9%–94.5%) to Est33 were chosen for further alignment. Ser^94^, Asp^205^, and His^233^ residues were indicated as the catalytic triad (Figure 1; Supplementary Figure S1), among which Ser^94^ was found in a pentapeptide sequence (GVSWG) conforming to a conserved motif (GXSXG) of esterases.

To confirm the importance of Ser^94^, Asp^205^, and His^233^, the corresponding codons were mutated. S94A, D205A, and H233A variants of Est33 were designed and expressed. These point substitutions all resulted in a complete loss of the enzyme activity (data not shown).

**Figure 2:**
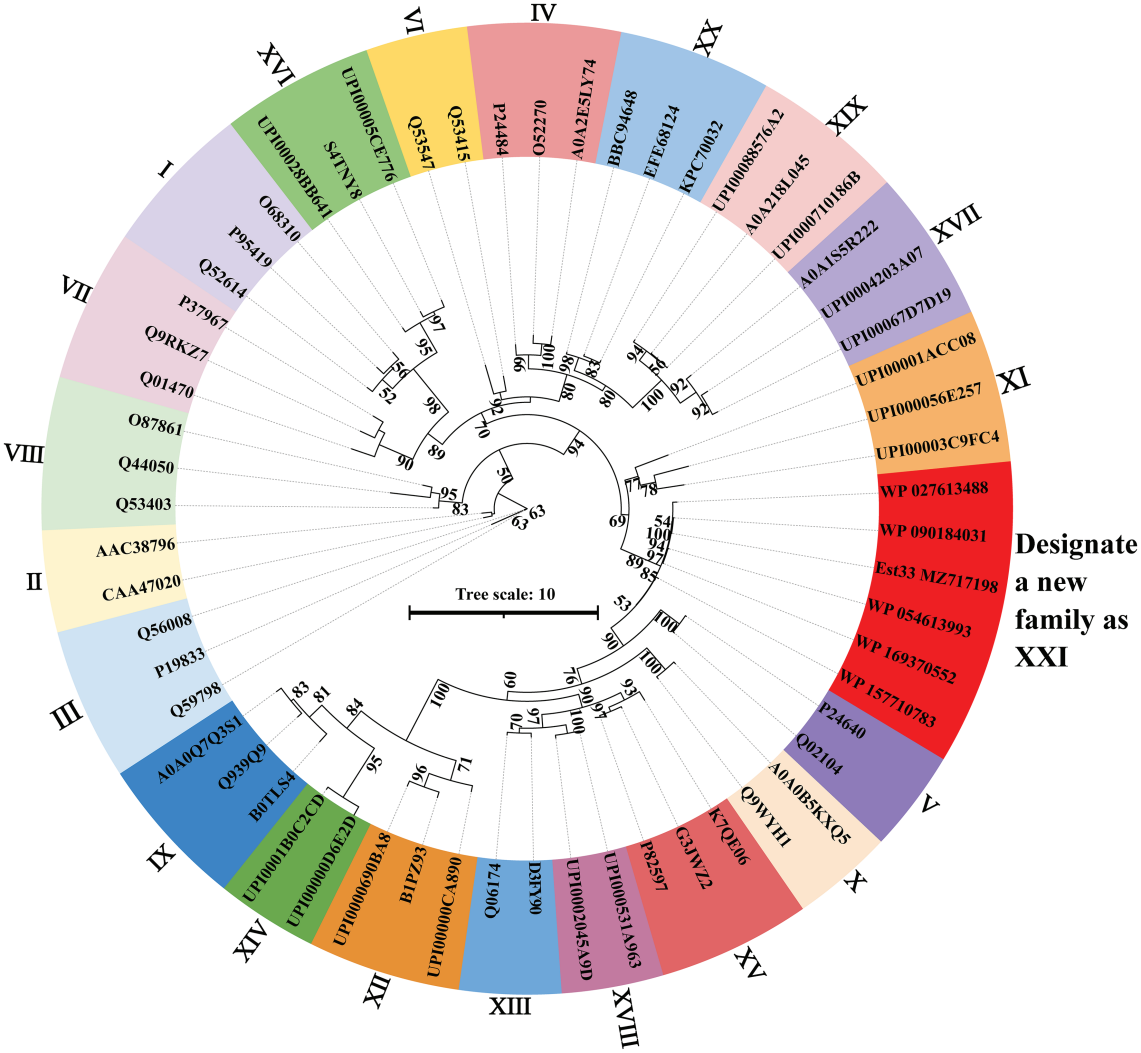
Phylogenetic analysis of Est33, its homologs, and other esterase families. The maximum likelihood phylogenetic tree was constructed using the IQ-TREE webserver with the auto substitution model and modified by iTOL v6. Bootstrap analysis of 1,000 replicates was conducted, and values above 50% are shown. Supplementary Table S1 shows the sources of all proteins shown.

SWISS-MODEL was used to predict the structure of Est33 and its homologs. The results indicated that Est33 and homologs possess a classical α/β hydrolase fold containing 9 β-sheet and 10 α-helices, with a highly conserved core structure and diverse loops (Figure 1; Supplementary Figure S1). Est33 and its closely related homologs form an independent group apart from the known 20 families in the constructed phylogenetic tree, indicating that Est33 represents a new family of esterase (Figure 2). This would make Est33 the first identified member of a novel esterase family, designated as family XXI.

### Preparation of Esterase Est33

To prepare the Esterase, est33 gene was ligated to the pET-22b vector and then transformed into *E. coli* BL21 (DE3) for expression. Nickel-affinity chromatography was used for purification against the 6*His tag contained in the recombinant protein. SDS-PAGE showed that the molecular weight of the recombinant enzyme Est33 was ~30 kDa, which was consistent with the theoretical molecular weight of 32.2 kDa (Supplementary Figure S2). N-termini sequencing of the purified enzyme revealed that the first amino acid residue Met^1^ was cut off during its expression in *E. coli*.

### Characteristics of Esterase Est33

**Figure 3:**
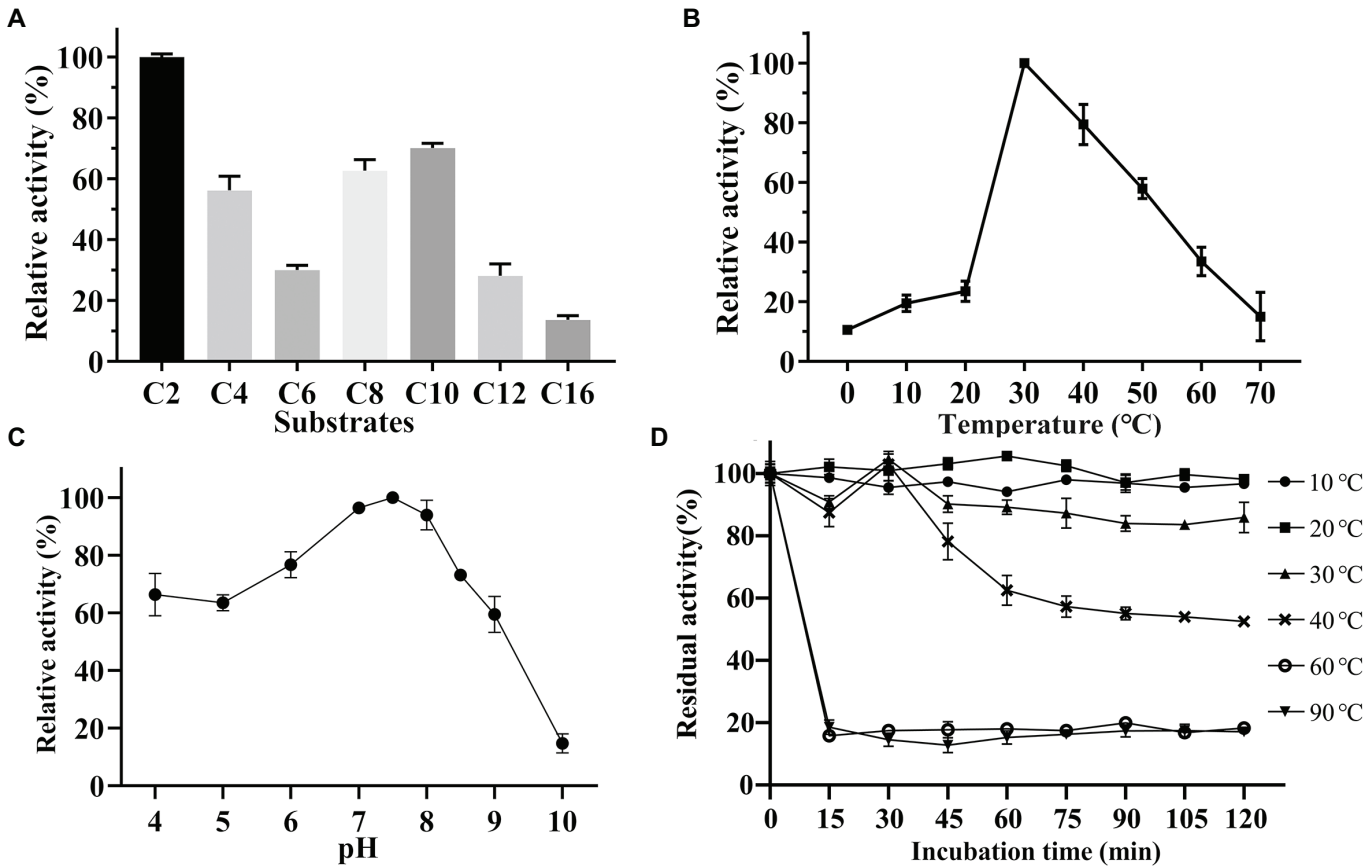
Biochemical characterization. **(A)** Substrate specificity of Est33. **(B)** Effect of temperature. **(C)** Effect of pH. **(D)** Enzyme thermostability. Results shown are means ±SD.

To evaluate the substrate specificity of Est33, *p*-nitrophenyl esters (*p*NP) with different acyl chain-lengths were employed as substrates. The enzyme displayed a maximum activity toward *p*NPC2, and it showed high activity against other short-chain esters. The ability of Est33 to hydrolyze substrates with relatively long acyl-chains (i.e., *p*NPC12 and *p*NPC16) was low (Figure 3A), indicating Est33 was an esterase rather than a lipase. The *K*m, *V*max, and *k*cat values of Est33 against *p*NPC2 were 275 μM, 1949 μM/min/mg, and 1.04 s^−1^, respectively, with a catalytic efficiency (*k*cat/*K*m) of 3.78 s^−1^ mM^−1^ (Supplementary Table S2).

The enzyme activity of Est33 at different temperatures and pH was measured using *p*NPC2 as the substrate in a standard reaction system (Figures 3B,C). The results showed that the optimum temperature and pH of enzyme Est33 were 30°C and 7.5, respectively. Est33 maintained more than 50% activity in the range of 30°C–50°C. Yet the activity dropped to 20% at 10°C–20°C, and 10% at 0°C and 70°C. Furthermore, enzyme thermal stability was evaluated by incubating Est33 at 30°C, 40°C, 60°C, and 90°C for different times (Figure 3D). The results showed that Est33 lost its activity very quickly after only 15 min at 60°C or 90°C. By contrast, Est33 could maintain more than 90% of its activity after incubation at 30°C and 50% at 40°C for 2 h, respectively.

### Effect of Inhibitors, Metal Ions, and Organic Solvents on Est33 Activity

The activity of Est33 was inhibited by ~80% in the presence of 10 mM PMSF (Table 1). The results suggest that Est33 is a serine hydrolase since PMSF is a serine modifying reagent. The latter is consistent with the behavior of the Ser-Asp-His catalytic triad identified by our initial sequence alignment (Figure 1) and results of site-directed mutagenesis (S94A) abolishing activity. In contrast, thiol directed agents such as DTT produced a marked (3-fold) increase in the enzymatic activity of Est33. Use of detergents (i.e., SDS and Tween80) and organic solvents generally inactivated the enzyme, especially 10% (w/v) SDS, which caused complete loss of activity (Table 1).

**Table 1:** Relative activity in the presence of various additives.^a^

Additives	Residual activity (%)	
EDTA	1 mM	103.15 ± 2.48
	10 mM	103.44 ± 5.28
PMSF	1 mM	57.31 ± 6.43
	10 mM	21.66 ± 7.98
DTT	1 mM	314.08 ± 31.41
	10 mM	382.91 ± 35.65
SDS	1% (w/v)	14.62 ± 1.05
	10% (w/v)	0
Tween-80	1% (w/v)	49.13 ± 0.91
	10% (w/v)	23.26 ± 3.12

a *Results were showed as the mean ± SD*.

**Figure 4:**
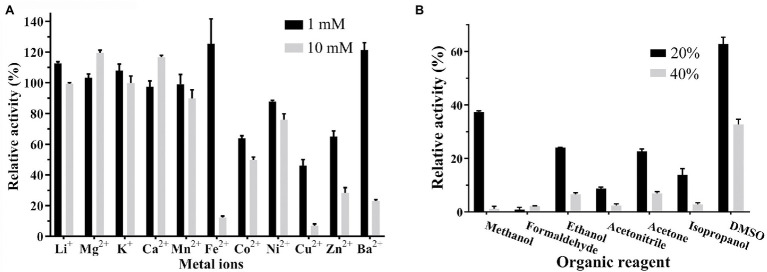
Effect of organic solvents and metal ions on the activity of Est33. **(A)** Organic solvents. **(B)** Metal ions. Results shown are means ±SD.

No effect of the metal ion chelator EDTA was observed on Est33 activity, suggesting that Est33 did not require metal ions for catalytic cycle. However, the presence of metal ions was found to have complex and varied effects on enzyme activity (Figure 4A). Among the metal ions tested, monovalent cations Li^+^ and K^2+^ had no effects. The divalent cations Cu^2+^, Zn^2+^, and Ba^2+^ had remarkable inhibitory effects, especially Fe^2+^, Cu^2+^, and Ba^2+^, which almost completely inhibited activity at a concentration of 10 mM. Yet, Co^2+^ and Ni^2+^ had medium inhibition effects. Surprisingly, when Fe^2+^was added at 1 mM, a slight activation of the enzyme activity was found. Similar dose response was obtained with Ba^2+^, where 1 mM concentrations slightly activated the enzyme and 10 mM almost completely inhibited activity.

As can be seen, the enzyme was inactivated almost completed by 20% or 40% formaldehyde. The tolerance of Est33 to acetonitrile was very low, and 40% acetonitrile inhibited Est33 almost completely. DMSO represented the only solvent tested that had only a modest effect on Est33 activity when used at high concentrations (i.e., 60% and 35% activity at 20% and 40% DMSO, respectively; Figure 4B).

## Discussion

Here, an esterase gene est33 was cloned from the genome of an Antarctic soil bacterial strain *P*. sp. E5-12 and then expressed in *E.coli* BL21 (DE3). The first 100 hits with highest identity were revealed to be all uncharacterized α/β hydrolases *via* protein BLAST analysis. The phylogenetic analysis revealed that Est33 and its closely related homologs formed a separate branch from the known 20 families (Figure 2). According to the BLAST results from the ESTHER database (a comprehensive classification and database system for α/β fold proteins; Lenfant et al., 2013), Est33 belonged to the PHA_depolymerase_arom family. Est33 along with the other 18 proteins from the PHA_depolymerase_arom family have a relatively low identity (32%–75%) compared to the sequences retrieved from the pBLAST in NCBI (Supplementary Figure S3). These results indicate that Est33 is the first member of a novel esterase family, which could be designated as family XXI.

The bacterial lipolytic enzymes have a catalytic triad composed of Ser, His, and Asp/Glu residues, and the catalytic Ser is located in the consensus pentapeptide motif Gly-x-Ser-x-Gly (GXSXG; Johan et al., 2021). The predicted catalytic triad of Est33 was formed by Ser^94^, Asp^205^, and His^233^ which play the role of nucleophile, charge-relay, and proton carrier, respectively (Figure 1; Supplementary Figure S1). The catalytic Ser94 was located in the pentapeptide motif GVSWG, which to our knowledge is first to be described in characterized esterases. A lot of novel esterases have been identified based on their similarities of conserved pentapeptide motif GXSXG and biochemical and structural characters, phylogenetic analysis (Johan et al., 2021). Substitution of any of the catalytic residues with alanine (A) caused a complete loss of enzymatic activity. Moreover, treatment of Est33 with PMSF inhibited enzymatic activity, consistent with the involvement of serine in the catalytic function of the enzyme (Curci et al., 2019; Kim et al., 2019).

Generally, cold-adapted enzymes have the characteristics of (i) higher catalytic efficiency at low temperature compared with their mesophilic analogues; (ii) lower optimum catalytic temperature; and (iii) lower thermal stability (Gerday et al., 1997). With an optimum temperature of 30°C, Est33 maintained about 10% of its activity at 0°C. Est33 lost its activity very quickly when incubated at 60°C or 90°C, while 2 h incubation at 40°C resulted in ~50% loss of its activity, indicating that it is a cold-adapted enzyme. Some cold-adapted esterases were previously isolated from Antarctic soil samples (Feller et al., 1991; Arpigny et al., 1993; Heath et al., 2009; Berlemont et al., 2013). Among them, the cold-active esterase MHlip has the same optimal reaction temperature as Est33 (Berlemont et al., 2013). However, MHlip lost its activity completely when the temperature reaches 65°C, while Est33 could maintain 30% of its activity at 60°C. Similarly, the cold-active esterase CHA3 was rapidly inactivated at 50°C (Heath et al., 2009), and EstA inactivated at 65°C (Cieslinski et al., 2007). Compared to these reported cold-active esterases, Est33 appears to be able to withstand higher temperatures.

A large number of cold-active enzymes are alkaline esterases and the optimum pH of approximately 8.0 (Luisa Tutino et al., 2009). Est33, like other cold-active esterases, has the maximum activity under slight alkaline conditions, having an optimal pH around 7.5 (Figure 3C). Moreover, Est33 has a broad of pH tolerance, with more than 70% enzyme activity remaining in the range of pH 6–8.5 and even more than 50% of its residual activity in the range of pH 4–9.

The activity of esterases is known to be affected either negatively or positively by different chemical compounds. In addition to the inhibition that was observed with PMSF, Est33 was found to be inhibited by ionic hydrophilic surfactant SDS and non-ionic hydrophilic surfactant Tween-80. However, EDTA had no effect on Est33 activity, suggesting that the enzyme does not contain metal ions critical to its structure and/or function (Table 1). In contrast however, the presence of metal ions was found to have appreciable effects on enzyme activity. For Est33, Co^2+^, Ni^2+^, Cu^2+^, and Zn^2+^ had inhibitory effects while Mg^2+^ and Ca^2+^ enhanced activity slightly (Figure 4A). Similar divergent effects with metal ions have been reported for other esterases in the literature (Lee et al., 2013).

All the organic reagents tested had inhibition effects on Est33 activity (Figure 4B). In the presence of 20% methanol, acetonitrile, or isopropanol, the residual enzyme activity was almost zero. Especially, formaldehyde caused completely inactivation even at low concentrations. By contrast, DMSO had a relatively small effect on the activity of Est33, which is similar to other reported esterases (Zhang et al., 2015; Curci et al., 2019; Tutuncu et al., 2019).

In summary, a novel esterase gene *est33* was cloned from Antarctic bacteria *Pseudomonas* sp. E5-12 and then expressed in *E. coli*. Ser^94^, Asp^205^, and His^233^ residues were identified to compose the catalytic triad. Sequence and phylogenetic analysis indicated that Est33 represents a new esterase family, which could be designated as family XXI. Biochemically characterization revealed that Est33 is a cold-adapted esterase, which displays maximum activity toward *p*NPC2 at 30°C and pH 7.5 with a thermal instability.

## Data Availability Statement

The data presented in the study are deposited in the GenBank repository, accession number MZ717198.

## Author Contributions

XL, MZ, and JC conceptualized and performed the experiments, evaluated the data, and drafted the manuscript together with SX and JB. RS, HH, and TW built the phylogenic tree and analyzed the data. All authors contributed to the article and approved the submitted version.

## Funding

This research was funded by the National Natural Science Foundation of China, grant numbers 31400002, 22171154, and 31370104; the Natural Science Foundation of Shandong Province, grant numbers ZR2020QB114 and ZR2020QB008; Youth Innovative Talents Recruitment and Cultivation Program of Shandong Higher Education; and Qilu University of Technology (Shandong Academy of Sciences) International Cooperation Fund, grant number QLUTGJHZ2018002.

## Conflict of Interest

The authors declare that the research was conducted in the absence of any commercial or financial relationships that could be construed as a potential conflict of interest.

## Publisher’s Note

All claims expressed in this article are solely those of the authors and do not necessarily represent those of their affiliated organizations, or those of the publisher, the editors and the reviewers. Any product that may be evaluated in this article, or claim that may be made by its manufacturer, is not guaranteed or endorsed by the publisher.

## Supplementary Material

The Supplementary Material for this article can be found online at: https://www.frontiersin.org/articles/10.3389/fmicb. 2022.855658/full#supplementary-material

## Footnotes

^1^ https://swissmodel.expasy.org/
