# Supplementary material for: Characterization of a Novel Esterase Est33 From an Antarctic Bacterium: A Representative of a New Esterase Family
Source: Front Microbiol. 2022 May 17;13:855658. doi: 10.3389/fmicb.2022.855658 (PMC9152352; doi:10.3389/fmicb.2022.855658)
Supplement: Supplementary file 1 [file Data_Sheet_1.docx]

Supplementary Material

## Supplementary Figures


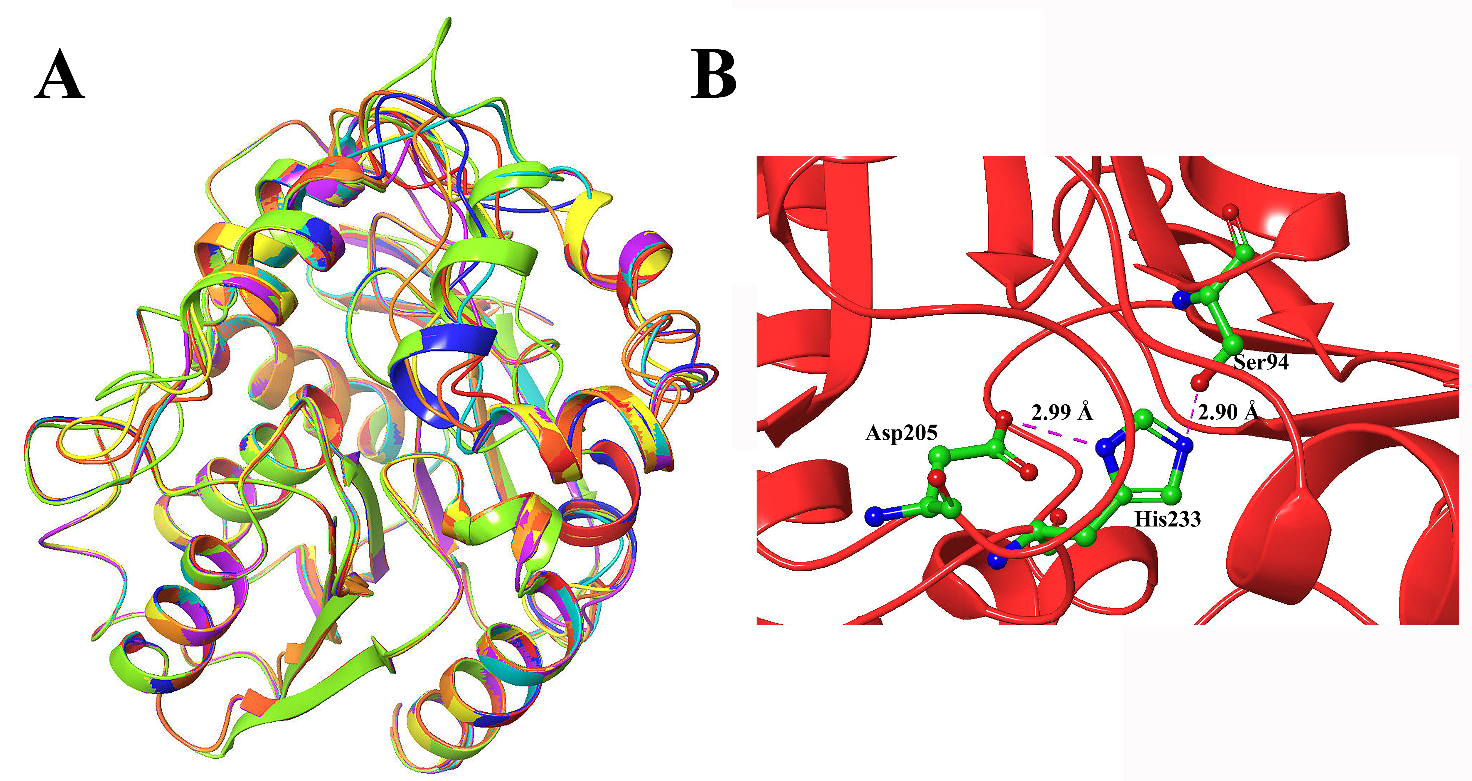


**Figure S1.** **Structures of new family esterase. a** Superimposition of the modeled structure of Est33 and its 7 homologs. The eight structures are colored in red (Est33), blue (WP_090184031), yellow (WP_217858764), plum (WP_157710783), teal (WP_083349625), yellow-green (WP_027613488), red-orange (WP_210710674), and orange (WP_008372319). **b** the structure of the active site of Est33, the catalytic triad, Ser^94^ (nucleophile), Asp^205^ (charge-relay), and His^233^ (proton carrier) are indicated, distances between catalytic Ser and His, His and Asp of Est33 were 2.90 Å, 2.99 Å, respectively.


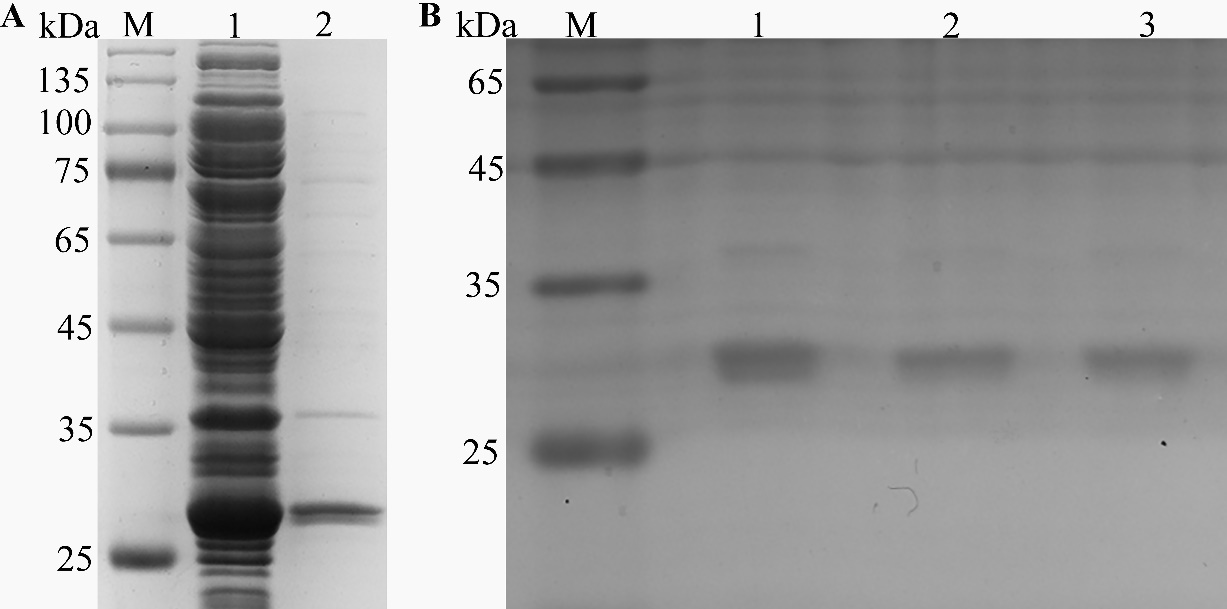


**Figure S2.** **SDS-PAGE analysis of Est33 and the variants.** Lane M, protein molecular mass marker; **a** Lane 1, total protein from *E. coli* with IPTG induction of Est33; Lane 2, purified Est33. b Lane 1-3, purified S94A, D205A, and H233A variants, respectively.


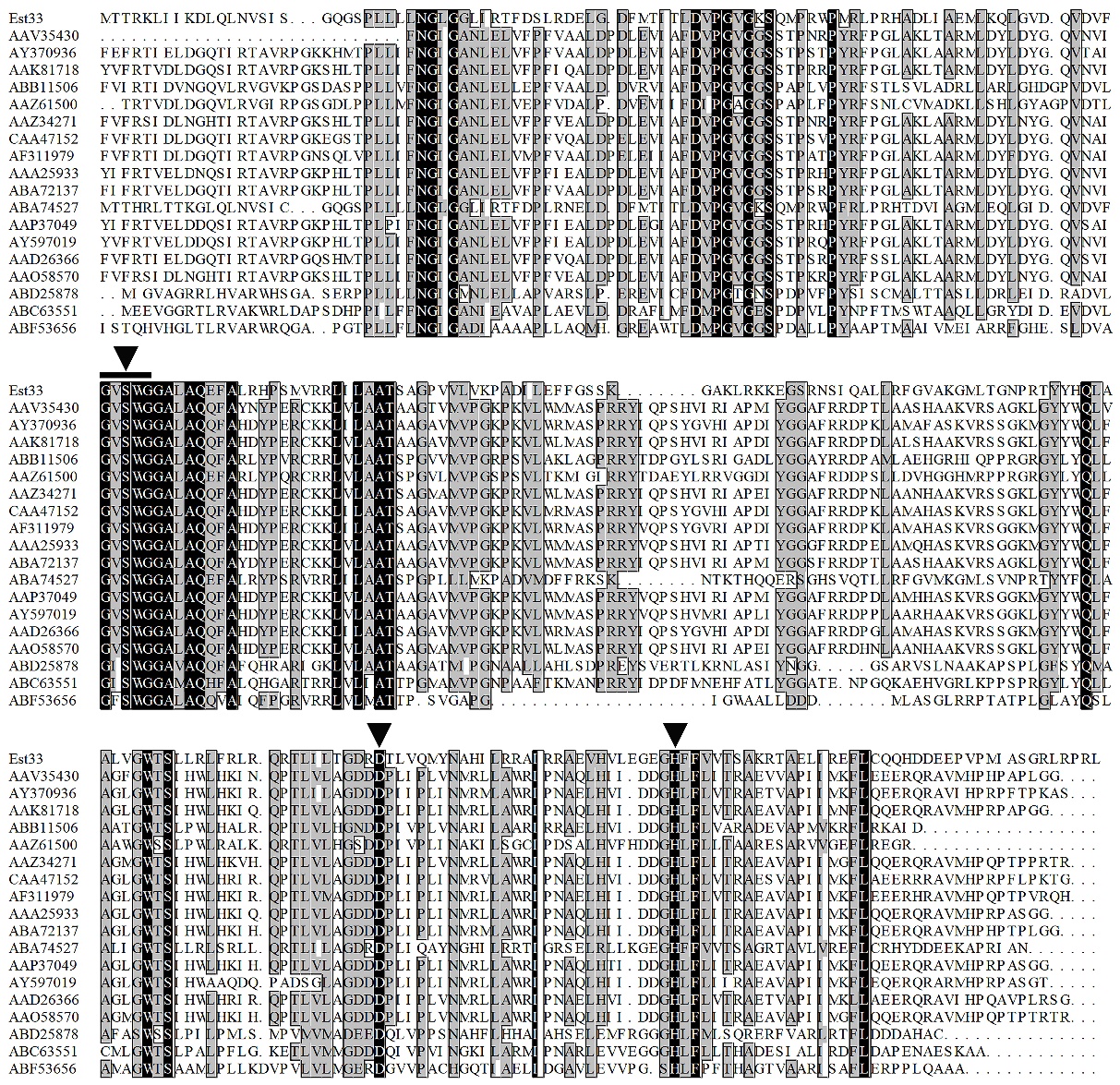


**Figure S3.** **Sequence alignment of Est33 and proteins from PHA_depolymerase_arom family.** Identical residues are shown in white on a black background, and similar residues (homology ≥75%) are on a grey background. Black triangle indicates residues forming the catalytic triad Ser, Asp and His. Black squares indicate conserved motifs.

## Supplementary Tables

**Table S1. Primers used in this study.**

| primers | sequence |
| --- | --- |
| est33F | AAGAAGGAGATATACATatgacaacgcgcaaactgatcattaa^a^ |
| est33R | TCGAGTGCGGCCGCAAGCTTgagcaggcgtggccttagcctgc |
| S94A-F | GATGTGTTCGGCGTG*GCT*TGGGGCGGTGCATTG^b^ |
| S94A-R | CAATGCACCGCCCCA*AGC*CACGCCGAACACATC |
| D205A-F | CTCACCGGCGACCGC*GCC*ACCCTGGTACAGATG |
| D205A-R | CATCTGTACCAGGGT*GGC*GCGGTCGCCGGTGAG |
| H233A-F | CTGGAGGGTGAGGGG*GCT*TTTTTCGTGGTCACC |
| H233A-R | GGTGACCACGAAAAA*AGC*CCCCTCACCCTCCAG |

^a^ Uppercase letters indicate the nucleotides pairing with the vector sequence. Lowercase letters indicate the esterase gene sequence. ^b^ The mutated site were shown in underline and italic. F, R in the name of primers means forward and reverse, respectively.

**Table S2.** **Kinetic parameters of esterase Est33 for *p*NPC2^a^.**

| Enzyme | *K_m_*  (μM) | *V*_max_  (μM/min/mg) | *k*_cat_  (s^−1^) | *k*_cat_/*K_m_*  (s^−1^mM^−1^) |
| --- | --- | --- | --- | --- |
| Est33 | 275±8 | 1949±44 | 1.04±0.02 | 3.78 |

^a^ Kinetic parameters were measured in triplicate using *p*NPC2 as substrate at 30 °C in Tris-HCl buffer (50 mM, pH 7.5). Results shown are means ±SD.

**Table S3.** Esterases sequences used in the phylogenetic analysis.

| **Organisms** | **UniProt or Genbank Accession No.** | **Family** |
| --- | --- | --- |
| *Proteus vulgaris* | Q52614 | Ⅰ |
| *Pseudomonas fluorescens* | O68310 | Ⅰ |
| *Pseudomonas aeruginosa* | P95419 | Ⅰ |
| *Salmonella typhimurium* | AAC38796.1 | Ⅱ |
| *Photorhabdus luminescens* | CAA47020.1 | Ⅱ |
| *Streptomyces albus* | Q59798 | Ⅲ |
| *Streptomyces* sp. | Q56008 | Ⅲ |
| *Moraxella* sp. (strain TA144) | P19833 | Ⅲ |
| *Dehalococcoidia* bacterium | A0A2E5LY74 | Ⅳ |
| *Pseudomonas* sp. B11-1 | O52270 | Ⅳ |
| *Moraxella* sp. (strain TA144) | P24484 | Ⅳ |
| *Psychrobacter immobilis* | Q02104 | Ⅴ |
| *Moraxella* sp. (strain TA144*)* | P24640 | Ⅴ |
| *Sulfolobus acidocaldarius* | O73957 | Ⅴ |
| *Arthrospira platensis* | Q53415 | Ⅵ |
| *Pseudomonas fluorescens* | Q53547 | Ⅵ |
| *Bacillus subtilis* (strain 168) | P37967 | Ⅶ |
| *Streptomyces coelicolor* (strain ATCC BAA-471) | Q9RKZ7 | Ⅶ |
| *Pseudarthrobacter oxydans* | Q01470 | Ⅶ |
| *Streptomyces anulatus* | O87861 | Ⅷ |
| *Pseudomonas fluorescens* | Q53403 | Ⅷ |
| *Arthrobacter globiformis* | Q44050 | Ⅷ |
| *Lysobacter* sp. Root494 | A0A0Q7Q3S1 | Ⅸ |
| *Paucimonas lemoignei* | Q939Q9 | Ⅸ |
| *Shewanella halifaxensis* (strain HAW-EB4) | B0TLS4 | Ⅸ |
| *Thermotoga* sp. RQ7 | A0A0B5KXQ5 | Ⅹ |
| *Thermotoga maritima* (strain ATCC 43589 ) | Q9WYH1 | Ⅹ |
| *Trichodesmium erythraeum* (strain IMS101) | UPI00003C9FC4 | Ⅺ |
| *Rhodopirellula baltica* (strain SH1) | UPI00001ACC08 | Ⅺ |
| *Colwellia psychrerythraea* | UPI000056E257 | Ⅺ |
| *Marinomonas* sp. MED121 | UPI0000690BA8 | Ⅻ |
| uncultured bacterium | B1PZ93 | Ⅻ |
| *Clostridium acetobutylicum* | UPI00000CA890 | Ⅻ |
| *Geobacillus stearothermophilus* | Q06174 | XIII |
| *Alkalihalobacillus pseudofirmus* (strain OF4) | D3FY90 | XIII |
| *Thermoanaerobacterium* | UPI0001B0C2CD | XIV |
| *Caldanaerobacter subterraneus* | UPI00000D6E2D | XIV |
| *Bacillus sp.* (strain H-257) | P82597 | XV |
| *Geobacillus thermodenitrificans* | G3JWZ2 | XV |
| uncultured bacterium | K7QE06 | XV |
| *Xanthomonas euvesicatoria* | UPI00005CE776 | XVI |
| *Saccharothrix espanaensis* | UPI00028BB641 | XVI |
| *Stenotrophomonas maltophilia* | S4TNY8 | XVI |
| *Arsenicicoccus* sp. oral taxon 190 | UPI00067D7D19 | XVII |
| *Dermatophilus congolensis* | UPI0004203A07 | XVII |
| *Janibacter* sp. R02 | A0A1S5R222 | XVII |
| *Solibacillus silvestris* | UPI0002045A9D | XVIII |
| *Lysinibacillus manganicus* | UPI000531A963 | XVIII |
| *Stenotrophomonas maltophilia* | UPI000710186B | XIX |
| *Stenotrophomonas maltophilia* | A0A218L045 | XIX |
| *Stenotrophomonas pavanii* | UPI00088576A2 | XIX |
| *Streptomyces* sp. NRRL WC-3753 | KPC70032.1 | XX |
| *Streptomyces viridosporus* ATCC 14672 | EFE68124.1 | XX |
| *Streptomyces rochei* | BBC94648.1 | XX |
| *Pseudomonas* sp. URIL14HWK12:I6 | WP 027613488 | XXI |
| *Pseudomonas arsenicoxydans* | WP 090184031 | XXI |
| *Pseudomonas* sp. In5 | WP 054613993 | XXI |
| unclassified *Pseudomonas* | WP 169370552 | XXI |
| *Pseudomonas* sp. PB120 | WP 157710783 | XXI |
| ***Pseudomonas* sp. E5-12** | **MZ717198 Est33** | **XXI** |
